# Systematic review and meta-analysis comparing microwave ablation vs. radiofrequency ablation for treatment of great saphenous vein reflux

**DOI:** 10.1590/1677-5449.202501832

**Published:** 2026-03-30

**Authors:** Luis Eduardo Rodrigues Sobreira, Vitor Kendi Tsuchiya Sano, Enrico Prajiante Bertolino, Marilia Pereira Costa, Altino Ono Moraes

**Affiliations:** 1 Universidade Federal do Pará – UFPA, Altamira, PA, Brasil.; 2 Universidade Federal do Acre – UFAC, Rio Branco, AC, Brasil.; 3 Universidade Estadual de Maringá - UEM, Maringá, PR, Brasil.; 4 Instituto de Assistência Médica ao Servidor Público Estadual de São Paulo – IAMSPE, São Paulo, SP, Brasil.

**Keywords:** varicose veins, meta-analysis, microwave ablation, radiofrequency catheter ablation, veias varicosas, metanálise, ablação por micro-ondas, ablação por cateter de radiofrequência

## Abstract

This study compares the outcomes of Endovenous Microwave Ablation (EMA) and Radiofrequency Ablation (RFA). A systematic search of the PubMed, Embase, Web of Sciences, and Scopus databases was conducted. Statistical analysis was performed with a random-effects model to compute mean differences (MD) and risk ratios (RR) with 95% confidence intervals. Three cohorts were included. Patients undergoing EMA showed a significant improvement in quality of life, with a reduction in Chronic Venous Insufficiency Questionnaire - 14 (CIVIQ-14) scores (MD −0.66; 95% CI −1.08 to −0.25; *P* < 0.01). Regarding length of stay, RFA patients may have experienced a slightly shorter recovery time than those in the EMA group (MD 0.12; 95% CI −0.01 to 0.30; *P* = 0.06). Lastly, phlebitis was slightly reduced in the EMA group, but this difference was also non-significant. Our findings revealed no significant differences between the two techniques.

## Resumo

Este estudo compara os desfechos da ablação endovenosa por micro-ondas (MWA) e da ablação por radiofrequência (ARF). Foi realizada uma busca sistemática nas bases de dados PubMed, Embase, Web of Science e Scopus. A análise estatística foi realizada com um modelo de efeitos aleatórios para calcular diferenças de média (DM) e razão de riscos com intervalos de confiança de 95%. Três coortes foram incluídas. Pacientes submetidos a MWA apresentaram melhora significativa na qualidade de vida, com redução nos escores do Chronic Venous Insufficiency Questionnaire - 14 (CIVIQ-14) (DM −0,66; IC95% −1,08 a −0,25; p < 0,01). O tempo de internação foi ligeiramente menor no grupo ARF (DM 0,12; IC95% −0,01 a 0,30; p = 0,06). Por último, a ocorrência de flebite foi ligeiramente menor no grupo MWA, mas esta diferença também não foi significativa. Nossas descobertas não revelaram diferenças significativas entre as duas técnicas.

## INTRODUCTION

Lower limb varicosities constitute a prevalent chronic venous disorder, with global estimates indicating rates as high as 57% in men and 73% in women, affecting a substantial number of individuals worldwide and exhibiting a marked predilection for females.^1-3^ These venous dilatations primarily arise due to valvular insufficiency, leading to subsequent venous hypertension, which can manifest clinically as pain, edema, and, at advanced stages, trophic skin changes or ulceration.^4^ Robust epidemiological evidence consistently demonstrates that the impact of this condition extends beyond symptomatic discomfort, significantly contributing to diminished quality of life and increased healthcare resource utilization across diverse socioeconomic settings; in the United States, venous leg ulcers affect roughly 2.2% of Medicare beneficiaries, imposing an annual payer burden of $14.9 billion.^5,6^ Given the persistent nature of lower extremity varicose veins and their potential for progressive complications, development and application of effective, minimally invasive therapeutic strategies is paramount to alleviate patient symptomatology, improve clinical outcomes, and mitigate the overall healthcare burden.

Contemporary advances in the management of great saphenous vein reflux have largely focused on endovenous ablation modalities, which have become a mainstay in current treatment paradigms. Of these, Endovenous Microwave Ablation (EMA) and Radiofrequency Ablation (RFA) have gained considerable traction owing to their feasibility in outpatient settings, obviating the need for hospital admission and consequently enhancing procedural efficiency and expediting patient recovery.^7,8^

The underlying physical mechanism of EMA involves an interaction between microwave electromagnetic fields (2.45 GHz) and biological tissues.^9^ EMA delivers energy through a catheter-based antenna positioned within the lumen of the target vein, producing rapid and controlled heating of the venous wall and adjacent tissues. This energy induces dielectric heating, resulting in coagulative necrosis of the vein wall, irreversible endothelial injury, and collagen denaturation, ultimately leading to vein collapse and fibrosis.^10^

In contrast, RFA delivers high-frequency alternating electrical current (0.5 - 0.75 MHz) via an intraluminal catheter, generating thermal energy through resistive heating of the vein wall.^11^ Moreover, RFA has been shown to achieve superior clinical outcomes compared to traditional invasive treatments.^12-14^

A direct comparative evaluation of EMA to RFA is crucial to ascertain the superior modality with respect to therapeutic efficacy, safety profile, and patient-reported outcomes. While existing literature suggests the effectiveness of both techniques, subtle yet potentially clinically relevant differences in recovery trajectories and post-procedural adverse events have been noted. Furthermore, a comprehensive assessment of the cost-effectiveness and long-term outcomes associated with each intervention is indispensable for evidence-based clinical decision-making and the judicious allocation of healthcare resources.

This novel systematic review with meta-analysis is designed to provide a comprehensive synthesis and comparison of the clinical outcomes after EMA and RFA for treatment of great saphenous vein reflux.

## METHODS

### Protocol, registration, and search strategy

This meta-analysis was conducted in January 2025 according to the guidelines established in the Preferred Reporting Items for Systematic Reviews and Meta-Analyses (PRISMA), following the Cochrane Collaboration methodological recommendations, and using the PubMed, EMBASE, Web of Science, and Scopus databases. Furthermore, the protocol for this study was prospectively registered on the Prospective International Registry of Systematic Reviews (PROSPERO) under identification number CRD42025648120, ensuring process transparency and methodological reproducibility and minimizing risk of bias. Registration on PROSPERO allows public access to the study objectives, eligibility criteria, and analytical strategies adopted, thereby strengthening the credibility and scientific rigor of this systematic review with meta-analysis. The search strategy employed Medical Subject Headings (MeSH) and Boolean operators (AND and OR) along with database-specific filters to optimize retrieval. Details of the search strategy are provided in Supplementary Table S1 (Supplementary Material).

All studies identified by the systematic search were imported into the Rayyan reference management platform.^15^ Duplicate records were detected and eliminated through a combination of automated procedures and manual verification using Zotero software. Subsequently, two independent reviewers (L.E.R.S. and M.P.C.) evaluated the titles and abstracts of the remaining unique studies against predetermined inclusion and exclusion criteria. Data extraction was carried out independently by the same reviewers using a standardized template, and methodological quality was appraised following established assessment guidelines. Any discrepancies in selection or data interpretation were resolved through consensus discussions and, when necessary, a third reviewer (V.K.T.S.) made the final adjudication.

### Eligibility criteria

In this study, the inclusion criteria were studies involving patients aged 18 years or older with a confirmed diagnosis of great saphenous vein reflux, established based on clinical manifestations and venous ultrasound. Eligible studies specifically evaluated microwave ablation in comparison to radiofrequency ablation. No language or location restrictions were applied for the selection. The individual inclusion and exclusion criteria for each study are listed in Supplementary Table S2.

We excluded studies with overlapping participant populations or periods, studies that did not use the interventions of interest, reviews, clinical guidelines, mouse studies, and in vitro experiments. Additionally, studies that did not report the relevant clinical outcomes necessary for the meta-analysis were also excluded.

### Data extraction

In summary, two independent reviewers (L.E.R.S. and E.P.B.) extracted key information from the selected articles, including authors, publication year, study design, and characteristics of the study groups, such as age, body mass index (BMI), great saphenous vein (GSV) diameter, and Clinical-Etiologic-Anatomic-Pathophysiologic (CEAP) class.

### Endpoints

The primary outcomes of interest were: (1) 12-month Chronic Venous Insufficiency Questionnaire - 14 (CIVIQ- 14) score (a shortened version of the CIVIQ-20); (2) 12-month Aberdeen Varicose Vein Questionnaire (AVVQ) score, which comprises items scored from 0 to 3 for symptom severity; (3) 12-month Venous Clinical Severity Score (VCSS), which comprises 10 items scored from 0 to 3 for symptom severity; and (4) total length of stay. The secondary outcomes of interest were: (1) ecchymosis; (2) induration; (3) paresthesia; (4) skin pigmentation; (5) great saphenous vein closure at 1 year; and (6) phlebitis.

### Risk of bias assessment

The Newcastle-Ottawa Scale (NOS) was used to assess study quality.^16^ Two reviewers (L.E.R.S. and M.P.C.) independently rated studies using the NOS, awarding up to nine stars based on selection, comparability, and outcome criteria. Disagreements were resolved through consensus.

### Statistical analysis

Treatment effects for dichotomous and continuous endpoints were pooled and reported as, respectively, risk-ratios (RR) and mean differences (MD) with 95% confidence intervals (CI). Heterogeneity was assessed using the Cochrane Q test and the I^2^ statistic. Significant heterogeneity was defined as a p-value > 0.10 or I^2^ > 25%. For the analyses, fixed-effects models using the Mantel-Haenszel and Inverse Variance methods were applied. Statistical analyses were performed with the “metafor” package, part of the R statistical software suite (version 4.2.3; R Foundation for Statistical Computing).^17,18^

## RESULTS

**Figure 1:**
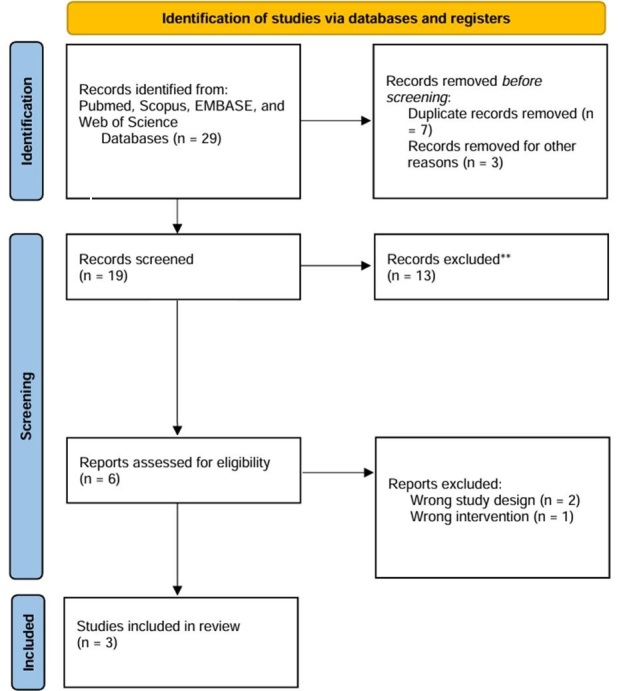
PRISMA flow diagram illustrating study screening and selection.

As detailed in Figure 1, the initial search yielded 29 results. After removing duplicate records and assessing the studies based on title and abstract, 19 studies remained for full-text review according to prespecified criteria. Of these, 3 studies comprising 847 patients were included, of whom, 416 (49.1%) underwent endovenous microwave ablation and 431 (50.8%) radiofrequency ablation. The study selection process is summarized in Figure 1.

### Study characteristics

Analysis of all included studies showed that mean age ranged from 52 to 58.1 years and 465 (54.9%) patients were female, while 382 (45.1%) were male. Across the studies, the body mass index ranged from 22.29 to 24.72 kg/m2, and GSV diameter ranged from 6.95 to 8.65 mm. The most prevalent CEAP classification was C4, with 282 (33%) cases, followed by C2 with 280 (32.7%). The follow-up period ranged from 20 to 41 months. There was no significant difference in comorbidities between the studies. The full study characteristics are presented in Table 1.

**Table 1:** Demographic and Clinical Data.

**Author**	**Country**	**Study period**	**Previous treatment**	**Study Design**	**Total patients - (EMA / RFA)**	**Gender (EMA/RFA)**		**Age, years (SD) - (EMA / RFA)**	**BMI, kg/m^2^ (SD) - (EMA / RFA)**	**CEAP class, n (%) - (EMA / RFA)**	**Diameter of GSV, mm (SD) - (EMA / RFA)**
						**Male, n (%)**	**Female, n (%)**				
Yang et al.^19^	China	2018 - 2021	No patients had previous treatment	Single-Center Retrospective Cohort	287 (142 / 145)	74 (52.1) / 81 (55.8)	68 (47.9) / 64 (44.2)	52.53 (11.16) / 52.11 (11.40)	22.46 (4.08) / 22.29 (3.93)	C2: 16 (11) / 17 (11.4) C3: 64 (43.8) / 66 (44.3) C4: 61 (41.8) / 60 (40.3) C5: 5 (3.4) / 6 (4)	8.65 (2.24) / 8.62 (2.13)
Zhang et al.^20^	China	2020 - 2022	No patients had previous treatment	Single-Center Retrospective Cohort	449 (209 / 240)	87 (41.6) / 88 (36.7)	122 (58.4) / 152 (63.3)	58.15 (12.43) / 57.88 (13.69)	23.53 (3.22) / 22.99 (3.45)	C2: 80 (38.3) / 90 (37.5) C3: 49 (23.4) / 74 (30.8) C4: 67 (32.1) / 61 (25.4) C5: 7 (3.3) / 9 (3.8) C6: 6 (2.9) / 6 (2.5)	6.95 (1.33) / 6.97 (1.58)
Zhao et al.^21^	China	2018 - 2020	The study included 6 patients with prior varicose veins treatment	Single-Center Retrospective Cohort	111 (65 / 46)	32 (49.2) / 20 (43.5)	33 (50.8) / 26 (56.5)	53.54 (11.98) / 52.02 (12.32)	24.72 (3.87) / 24.27 (3.38)	C2: 18 (27.7) / 11 (23.9) C3: 12 (18.4) / 15 (32.6) C4: 22 (33.8) / 11 (23.9) C5: 6 (9.2) / 3 (6.5) C6: 7 (10.7) / 6 (13)	8.33 (1.78) / 8.44 (0.76)

Legend: CEAP = Clinical-Etiologic-Anatomic-Pathophysiologic Classification; EMA = Endovenous Microwave Ablation; RFA = Radiofrequency Ablation; SD = Standard Deviation; BMI = Body mass index; GSV = Great Saphenous Vein.

### Pooled analysis of the outcomes

**Figure 2:**
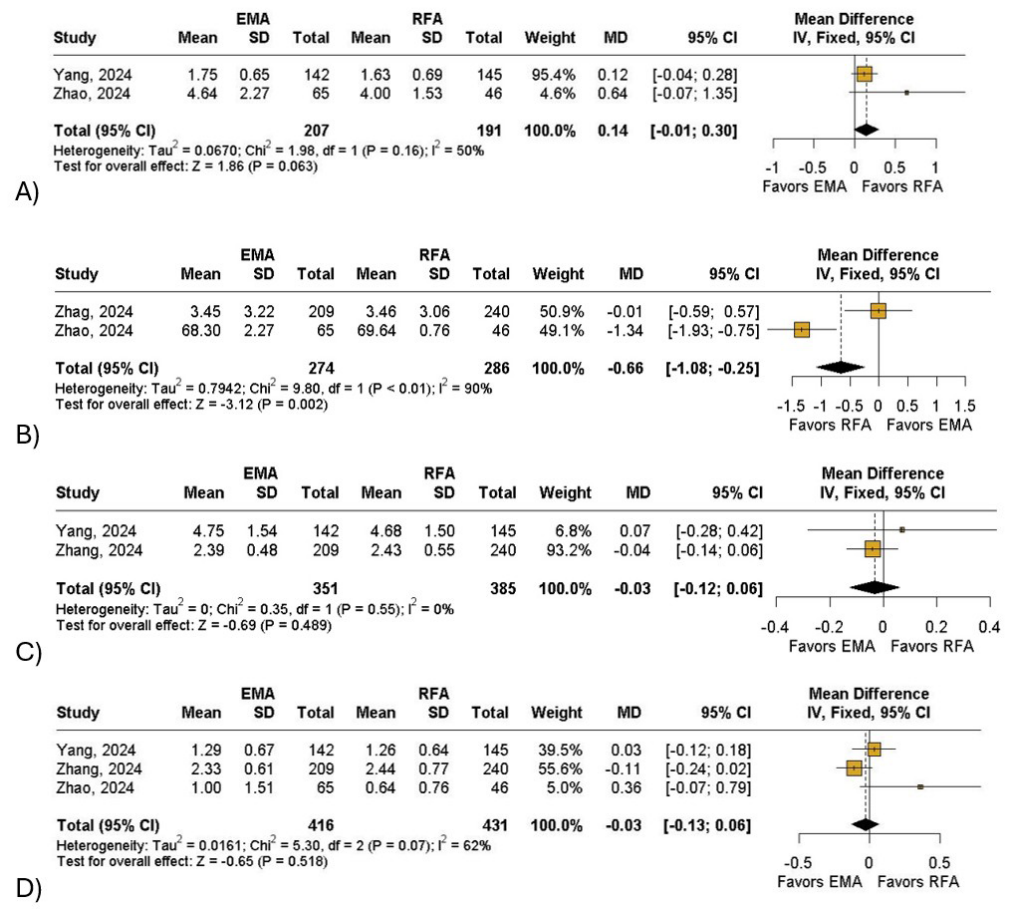
Forest plot of primary outcomes. (A) Chronic Venous Insufficiency Questionnaire; (B) Aberdeen Varicose Vein Questionnaire; (C) Venous Clinical Severity Score; (D) Total Length of Stay. Legend: EMA = endovenous microwave ablation; SD = standard deviation; RFA = radiofrequency ablation; MD = mean difference; CI = confidence interval; IV = inverse variance.

**Figure 3:**
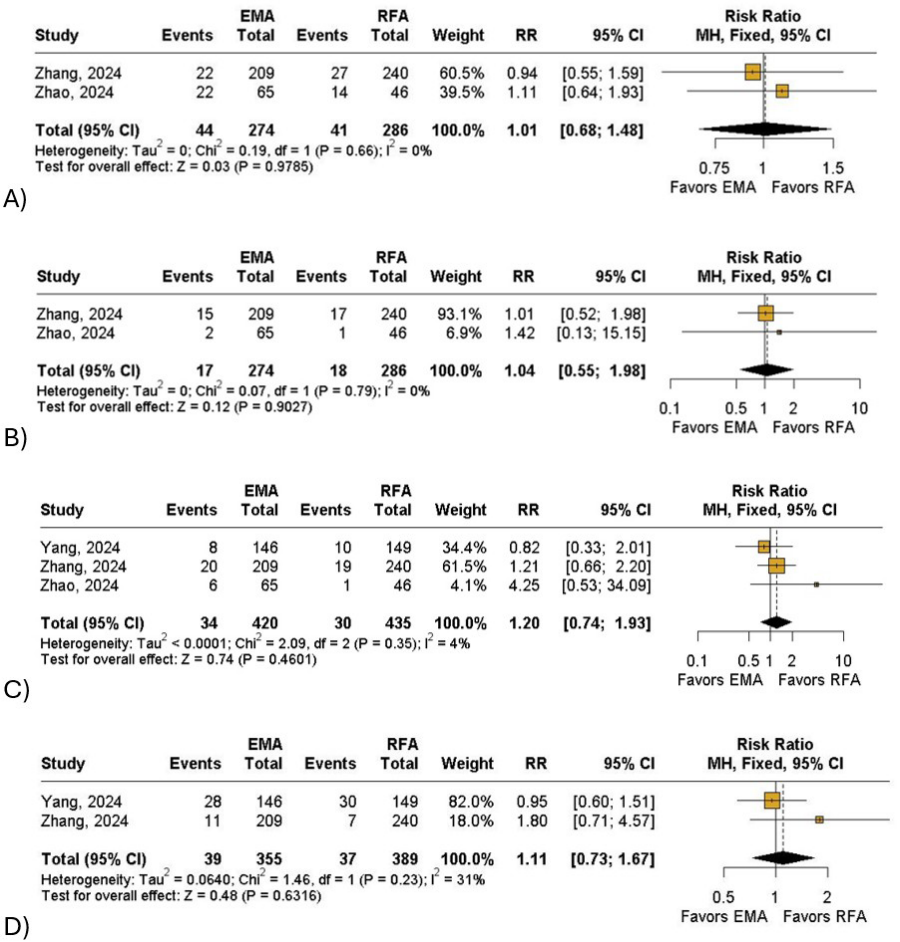
Forest plot of secondary outcomes. (A) Ecchymosis; (B) Induration; (C) Paresthesia; (D) Skin pigmentation. Legend: EMA = endovenous microwave ablation; RFA = radiofrequency ablation; RR = risk ratio; CI = confidence interval; MH = Mantel-Haenszel; df = degrees of freedom

**Figure 4:**
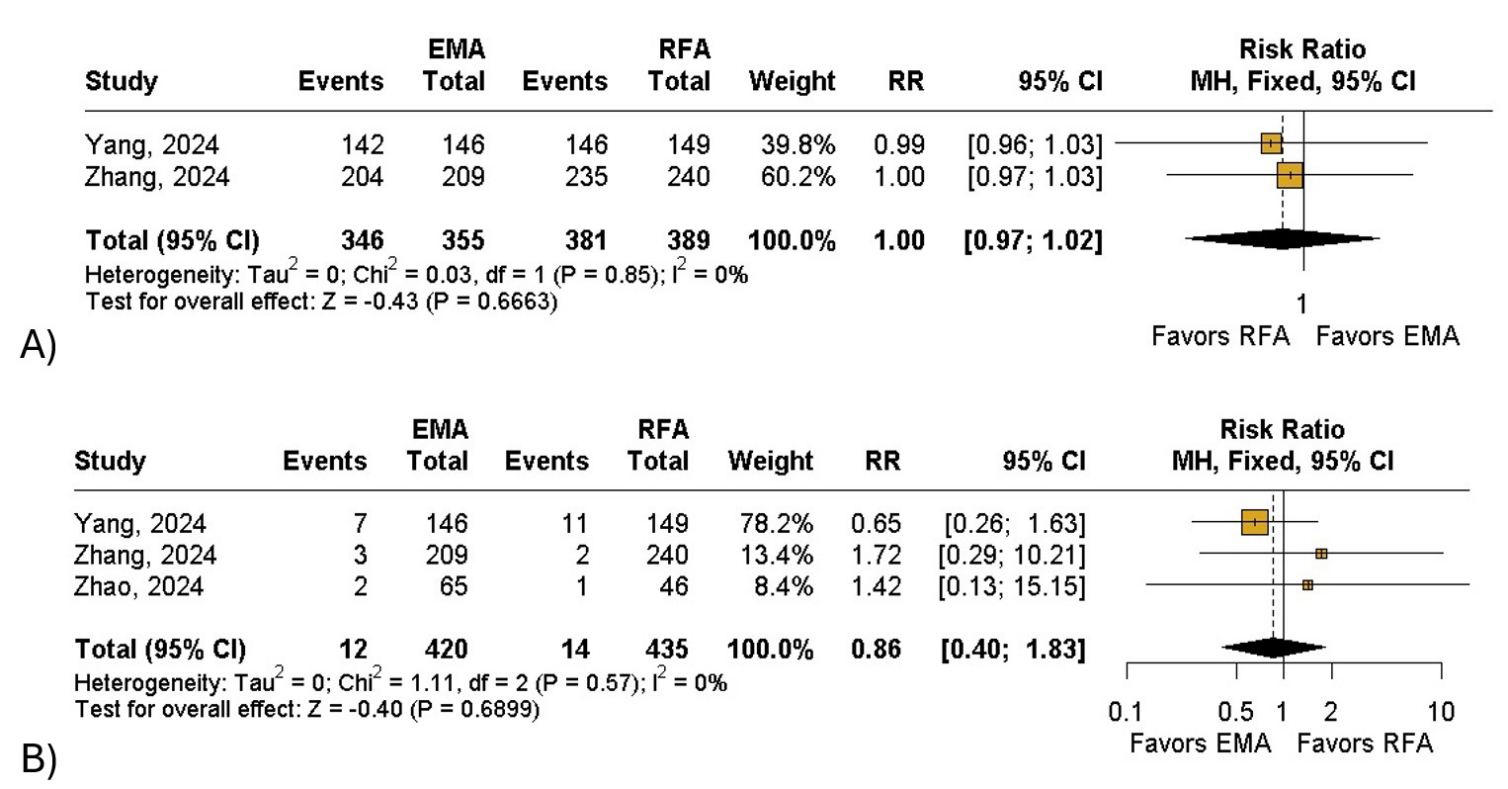
Forest plot of secondary outcomes. (A) Great Saphenous Vein Total Closure; (B) Phlebitis. Legend: EMA = endovenous microwave ablation; RFA = radiofrequency ablation; RR = risk ratio; CI = confidence interval; MH = Mantel-Haenszel; df = degrees of freedom.

Overall, patients who underwent EMA had a significant decrease in CIVIQ-14 score (MD: −0.66; 95% CI: −1.08 to −0.25; I^2^= 90%; *P* < 0.01; Figure 2A) when compared to the RFA group. The EMA group was also associated with a non-significant reduction in AVVQ (MD: −0.03; 95% CI: −0.12 to 0.06; I^2^= 0%; *P* = 0.48; Figure 2B) and VCSS scores (MD: −0.03; 95% CI: −0.13 to 0.06; I^2^= 62%; *P* = 0.51; Figure 2C) when compared with the control group. In terms of length of stay, patients who underwent RFA might have shorter recovery time when compared to those in the EMA group (MD: 0.12; 95% CI: −0.01 to 0.30; I^2^= 50%; *P* = 0.06; Figure 2D). Furthermore, the RFA group had a non-significant reduction in ecchymosis (RR: 1.01;95% CI: 0.68 to 1.48; I^2^= 0%;*P* = 0.97; Figure 3A), induration (RR: 1.04; 95% CI: 0.55 to 1.98; I^2^= 0%; *P* = 0.90;Figure 3B), paresthesia (RR: 1.20;95% CI: 0.74 to 1.93; I^2^= 4%; *P* = 0.46; Figure 3C) and skin pigmentation (RR: 1.11; 95% CI: 0.73 to 1.67; I^2^= 31%; *P* = 0.63; Figure 3D) and a non-significant increase of great saphenous vein closure at 1 year (RR: 1.00; 95% CI: 0.97 to 1.02; I^2^= 0%; *P* = 0.66; Figure 4A) when compared with the intervention group. Finally, patients treated with EMA had a non-significant reduction in phlebitis (RR: 0.86; 95% CI: 0.40 to 1.83; I^2^= 0%; *P* = 0.68; Figure 4B) compared with the other group.

### Quality assessment

Since no RCTs were included in this synthesis, all assessments were conducted using the Newcastle-Ottawa Scale. Overall, all included studies were deemed of good quality. The individual assessments are shown in Supplementary Table S3.

## DISCUSSION

This is the first meta-analysis to directly compare EMA and RFA. In this study, which included 847 patients from three retrospective cohort studies, RFA demonstrated an improvement in quality of life by significantly reducing CIVIQ-14 scores. However, EMA slightly decreased the AVVQ score, though without statistical significance. Conversely, although no statistically significant differences were observed in adverse event outcomes, EMA was associated with a longer hospital stay and an increased risk of paresthesia.

Endovenous microwave ablation is the latest development in treatment of varicose veins in the legs. Similar to other endovenous ablation techniques, it uses heat to permanently eliminate refluxing veins.^22^ In this technique, a microwave ablation catheter is percutaneously inserted into the varicose vein, where the antenna emits penetrating microwave energy that causes polar molecules in the vascular tissue to vibrate at high frequency under the influence of the microwave field, generating heat that leads to thermal ablation of the vein.^23^ Hence, to maintain the pullback speed of 4-9 s/cm, power settings of 35-70 W are needed.^24^

Radiofrequency ablation treats varicose veins by delivering controlled thermal energy to the vein wall, leading to endothelial injury, collagen denaturation, and subsequent vein closure. The mechanism involves heating the vein wall via resistive heating, causing structural damage that initiates fibrosis and occlusion of the treated vein.^25^ Power settings of 18-20W and catheter withdrawal speed of approximately 1.5s/cm are recommended with this technique.^26^

Unlike radiofrequency ablation, in which temperatures are up to 120 °C, the temperature attained in EMA is around 50-80 °C, significantly lowering the risks of skin burns and other dermatological damage.^27-29^ The main goal of varicose vein treatment is improvement of patients’ quality of life.

Recently, a meta-analysis conducted by Kalaij et al.^30^ compared the safety and efficacy of EMA vs. endovenous laser ablation (EVLA). The authors found that EMA had a similar Aberdeen score compared to EVLA (MD 0.59; 95% CI −2.37-1.20), EMA had a similar probability of paresthesia incidence compared to EVLA (OR 0.61; 95% CI 0.20-1.85), and EMA had a similar probability of skin burn incidence compared to EVLA (OR 1.01; 95% CI 0.46-2.22). Our study found similar results when comparing EMA vs. RFA.

Regarding quality of life, there was no statistically significant difference in AVVQ scores between the groups (MD: −0.03; 95% CI: −0.12 to 0.06; *P* = 0.48). This indicates that neither EMA nor RFA was more effective in reducing the impact of varicose veins on patients’ daily lives. However, EMA showed a significant reduction in the CIVIQ-14 score (MD: −0.66; 95% CI: −1.08 to −0.25; *P* = 0.002). The low CIVIQ-14 scores indicate a greater negative impact of chronic venous disease on patients’ quality of life, reflecting more pronounced symptoms and limitations in daily activity.

In terms of efficacy, both treatment modalities demonstrated comparable effectiveness for reducing VCSS scores (MD: −0.03; 95% CI: −0.13 to 0.06; *P* = 0.51) and achieving great saphenous vein closure at 1 year (RR: 1.00; 95% CI: 0.97 to 1.02; *P* = 0.66). Regarding safety, both groups showed similar outcomes, with no significant differences observed in the incidence of adverse events, including ecchymosis (RR: 1.01; 95% CI: 0.68 to 1.48; *P* = 0.97), induration (RR: 1.04; 95% CI: 0.55 to 1.98; *P* = 0.90), paresthesia (RR: 1.20; 95% CI: 0.74 to 1.93; *P* = 0.46), skin pigmentation (RR: 1.11; 95% CI: 0.73 to 1.67; *P* = 0.63), and phlebitis (RR: 0.86; 95% CI: 0.40 to 1.83; *P* = 0.68).

Our study has several limitations. First, we only included a limited number of non-randomized studies in our analysis because of the lack of trials comparing EMA vs. RFA. Second, the study by Yang et al.^19^ used different EMA devices for thermal ablation. Finally, the Zhao et al.^21^ study included some patients who had been treated previously for varicose veins. All of these limitations may have impacted our final results.

## CONCLUSION

This systematic review and meta-analysis evaluated the efficacy and safety of EMA compared to RFA for the treatment of great saphenous vein reflux. Our findings revealed no significant differences between the two techniques. Future randomized multicenter trials conducted globally are warranted to better determine which approach offers superior safety and effectiveness for management of great saphenous vein reflux.

## Data Availability

All data generated or analyzed are included in this article and/or in the supplemental material.

## Supplementary Material

Supplementary material accompanies this paper.

Supplementary Table S1. Search Strategies

Supplementary Table S2. Inclusion and exclusion criteria of included studies

Supplementary Table S3. Methodological quality summary using Newcastle Ottawa Scale.

This material is available as part of the online article from https://doi.org/10.1590/1677-5449.202501832
