# Supplementary material for: Systematic review and meta-analysis comparing microwave ablation vs. radiofrequency ablation for treatment of great saphenous vein reflux
Source: J Vasc Bras. 2026 Mar 30;25:e20250183. doi: 10.1590/1677-5449.202501832 (PMC13078176; doi:10.1590/1677-5449.202501832)
Supplement: Supplementary Table S1. [file jvb-25-e20250183-suppl01.pdf]

Supplementary Material

Supplementary Table S1. Search Strategies

Database	Search Strategy
**PubMed**	((Microwave Ablation) AND ((((((Ablation, Radiofrequency) OR (Radio-Frequency Ablation)) OR (Ablation, Radio-Frequency)) OR (Radio Frequency Ablation)) OR (Ablation, Radio Frequency)) OR (Radiofrequency Ablation))) AND (((((Varicose Vein) OR (Veins, Varicose)) OR (Vein, Varicose)) OR (Varices)) OR (Varix))
**Embase**	("Microwave Ablation":ti,ab,kw) AND ("Ablation, Radiofrequency":ti,ab,kw OR "Radio-Frequency Ablation":ti,ab,kw OR "Ablation, Radio-Frequency":ti,ab,kw OR "Radio Frequency Ablation":ti,ab,kw OR "Ablation, Radio Frequency":ti,ab,kw OR "Radiofrequency Ablation":ti,ab,kw) AND ("Varicose Vein":ti,ab,kw OR "Veins, Varicose":ti,ab,kw OR "Vein, Varicose":ti,ab,kw OR "Varices":ti,ab,kw OR "Varix":ti,ab,kw)
**Web of Science**	(TS=("Microwave Ablation")) AND (TS=("Ablation, Radiofrequency") OR TS=("Radio-Frequency Ablation") OR TS=("Ablation, Radio-Frequency") OR TS=("Radio Frequency Ablation") OR TS=("Ablation, Radio Frequency") OR TS=("Radiofrequency Ablation")) AND (TS=("Varicose Vein") OR TS=("Veins, Varicose") OR TS=("Vein, Varicose") OR TS=("Varices") OR TS=("Varix")))

**Scopus**	(TITLE-ABS-KEY("Microwave Ablation") AND (TITLE-ABS-KEY("Ablation, Radiofrequency") OR TITLE-ABS-KEY("Radio-Frequency Ablation") OR TITLE-ABS-KEY("Ablation, Radio-Frequency") OR TITLE-ABS-KEY("Radio Frequency Ablation") OR TITLE-ABS-KEY("Ablation, Radio Frequency") OR TITLE-ABS-KEY("Radiofrequency Ablation")) AND (TITLE-ABS-KEY("Varicose Vein") OR TITLE-ABS-KEY("Veins, Varicose") OR TITLE-ABS-KEY("Vein, Varicose") OR TITLE-ABS-KEY("Varices") OR TITLE-ABS-KEY("Varix")))
