# Supplementary material for: Systematic review and meta-analysis comparing microwave ablation vs. radiofrequency ablation for treatment of great saphenous vein reflux
Source: J Vasc Bras. 2026 Mar 30;25:e20250183. doi: 10.1590/1677-5449.202501832 (PMC13078176; doi:10.1590/1677-5449.202501832)
Supplement: Supplementary Table S2. [file jvb-25-e20250183-suppl02.pdf]

**Supplementary Material**

**Supplementary Table S2.** Inclusion and exclusion criteria of included studies

| Study | Inclusion Criteria | Exclusion Criteria |
| --- | --- | --- |
| Yang, 2024 | 1) diagnosis of lower limb VVs based on clinical manifestations and a duplex ultrasound examination;<br>2) GSV reflux >0.5 seconds;<br>3) deep vein patency;<br>4) Clinical, Etiology, Anatomy, and Pathophysiology classification between C2 and C5; 5) complete clinical data;<br>6) no surgical contraindications. | Patients with symptomatic peripheral arterial disease, deep venous thrombosis in the lower extremities, those who had undergone previous VV treatment in the same limb, and those who did not wish to participate were excluded. |
| Zhang, 2024 | 1) Saphenous femoral venous valve regurgitation time greater than 0.5 seconds confirmed by Doppler ultrasound;<br>2) Great saphenous vein diameter between 3 mm and 12 mm confirmed by Doppler ultrasound;<br>3) Clinical symptoms of varicose veins in the lower | Patients were excluded if they had:<br>1) great saphenous vein thrombosis;<br>2) deep vein thrombosis or a history of deep vein thrombosis and pulmonary embolism;<br>3) prior surgery or sclerotherapy treatment for lower limb |

| | | |
|---|---|---|
| | extremities with a CEAP classification of C2 to C6 requiring treatment of the main saphenous vein. | varicose veins;<br>4) pregnancy or lactation, or plans to become pregnant within one year;<br>5) lower limb artery stenosis or occlusion. |
| Zhao, 2024 | The criteria for inclusion were as follows: symptomatic LLVVs (CEAP class C2-C6); GSV, SSV, or ASV incompetence, also validated as a reflux time >=0.5 seconds on Doppler ultrasound; pathologic PVs, also validated as a reflux time >=0.5 seconds and a diameter >=3.5 mm underneath a healed or active ulcer on ultrasound; and use of EMA or RFA for the treatment of LLVVs for symptoms such as swelling, pain, stasis dermatitis, and stasis ulcer. | The criteria for exclusion were as follows:<br>1) Suspected or proven deep vein thrombosis or occlusion;<br>2) Deep vein reflux to a distal limb;<br>3) Contraindications to anesthesia and surgery;<br>4) Refusal to participate in the investigation. |
