# Supplementary material for: Systematic review and meta-analysis comparing microwave ablation vs. radiofrequency ablation for treatment of great saphenous vein reflux
Source: J Vasc Bras. 2026 Mar 30;25:e20250183. doi: 10.1590/1677-5449.202501832 (PMC13078176; doi:10.1590/1677-5449.202501832)
Supplement: Supplementary Table S3. [file jvb-25-e20250183-suppl03.pdf]

Supplementary Material

Supplementary Table S3. Methodological quality summary using Newcastle Ottawa Scale.

Meta-data			Methodology	Newcastle-Ottawa Scale			
**Author**	**Publication Date**	**Origin**	**Study design***	**Selection**	**Comparability**	**Exposure**	**Quality**
Yang	2024	China	--	☆☆☆	☆☆☆	☆☆☆	9
Zhang	2024	China	--	☆☆	☆☆☆	☆☆☆	8
Zhao	2024	China	--	☆☆☆	☆☆☆	☆☆☆	9

Abbreviations: *Study design: Prospective (+), Retrospective (−); single center (−), multicenter (+), Maximum quality score = 9; 0–7 points were considered lower quality, and 8–9 points were considered as higher quality
